# Detection of Fibrinogen and Coagulation Factor VIII in Plasma by a Quartz Crystal Microbalance Biosensor

**DOI:** 10.3390/s130606946

**Published:** 2013-05-24

**Authors:** Chunyan Yao, Ling Qu, Weiling Fu

**Affiliations:** 1 Department of Laboratory Medicine, Southwest Hospital, the Third Military Medical University, Chongqing 400038, China; 2 Department of Laboratory Medicine, Xijing Hospital, the Fourth Military Medical University, Xi'an 710032, China; E-Mail: qulingcc@163.com

**Keywords:** fibrinogen, coagulation factor VIII, quartz crystal microbalance, biosensor

## Abstract

A quartz crystal microbalance (QCM) biosensor with nanogram sensitivity has been constructed through a reasonable designing and biological processing of the piezoelectric quartz crystals. Due to its highly sensitivity, real time detection and low cost, the proposed QCM biosensor has a promising potential in blood coagulation research. In the current study, the QCM biosensor was used to determine the activated partial thromboplastin time (APTT) for 120 anticoagulated plasma specimens. A good linear relationship was found in a double-logarithmic plot of APTT *versus* fibrinogen concentration in the range of 1.58–6.30 g/L. For factor VIII, the detection range by the QCM biosensor is 0.0185–0.111 mg/L. The QCM biosensor results were compared with those obtained by commercial optical coagulometry and a good agreement (correlation coefficient is 0.949 for fibrinogen, and 0.948 for factor VIII) was reached. Furthermore, the QCM determination can be completed within 10 min. Our study suggested that the proposed QCM biosensor could provide for more convenient and time saving operations, which may be useful in clinical situations for rapid monitoring of anticoagulant therapy using small volume (20 μL) plasma specimens.

## 1. Introduction

Early and reliable diagnosis of haemostatic disorders increases the possibility of successful medical treatment. The analysis of blood coagulation factors is widely performed in the clinical laboratory because it plays a central role in providing important information to the physician so as to minimize the patient's risk. Among these tests, fibrinogen and blood coagulation factor VIII (FVIII) are often analyzed in clinical laboratories to monitor the extent of the introduced anticoagulation. Fibrinogen, which plays an important role in the blood coagulation cascade, can be activated by thrombin and polymerized insoluble fibrin, a main component of clots [1]. Meanwhile, coagulation FVIII participates in the blood clotting cascade as a cofactor of FIXa in the activation of FX. The congenital deficiency or abnormality of coagulation FVIII results in a severe bleeding disorder, namely, hemophilia A, which is potentially life-threatening and crippling [2,3]. Low plasma levels of coagulation FVIII leads to a tendency towards clotting inefficiencies, whereas high plasma levels of coagulation FVIII can result in various thrombotic diathesis, such as deep vein thrombosis [4-6]. Rapid and accurate detection of fibrinogen and FVIII is valuable to evaluate response to therapy and is available for identifying risk control for patients. However, the current laboratory test for fibrinogen and coagulation FVIII is time consuming and expensive. Therefore, a rapid and convenient method for monitoring concentrations of fibrinogen and FVIII is urgent for both physicians and patients.

Activated partial thromboplastin time (APTT) is routinely used for monitoring anticoagulation therapy in clinical practice. It is also useful for the detection of the abnormal factors I, II, V, VIII, IX, X, XI and XII [7]. Normal APTT times require the presence of the following coagulation factors: I, II, V, VIII, IX, X, XI, and XII. Deficiencies of factors VIII may lead to a prolonged APTT. Since the APTT duration was decided by concentration of factor VIII, a short reaction time should be expected with the high concentration of factor VIII. Thus, the concentration of factor VIII can be calculated from the APTT time.

Blood coagulation kinetics and clot character are studied in various ways such as spectrophotometry [8], quartz crystal microbalance (QCM) [9], magnetoelastic sensor [10], surface plasma resonance (SPR) [11-13] and free oscillation rheometry [14]. The optical coagulometer based on stop-vibrating detection, which is more accurate in determining the coagulation end-point, has also been employed for blood coagulation measurement [15] and is commonly used for laboratory hemostatic surveys. However, the optical coagulometer needs expensive equipment and thus the detection cost is high for the patients. The QCM biosensor has turned out to be a very useful alternative for determination of surface induced blood coagulation, since fibrin formation can be detected *in situ* and in real time on the electrode surface [16,17]. The classical QCM technique has been previously used to determine the concentration of fibrinogen [18,19], activity of blood coagulation factors [20], and coagulation time [21-23] with the advantages of real time output, label-free and high sensitivity. The frequency shifts give information about the general clotting kinetics, such as time of onset and fibrin deposition rate.

We have successfully developed a QCM biosensor for high sensitivity detection of protein and nucleic acid [24,25]. In the current paper, we used the QCM biosensor to detect the concentrations of fibrinogen and coagulation FVIII in clinical plasma specimens, and compared the QCM results with those measured by the optical coagulometer (OC) method which is commonly used in the clinical laboratory. We hope to develop a new detection technique based on the QCM method for accurate and fast detection of fibrinogen and coagulation FVIII. In order to explore its feasibility for clinical application, a series of investigations were conducted to detect fibrinogen and coagulation FVIII after optimizing the parameters. The proposed QCM biosensor proved to be an accurate, fast and economic method for fibrinogen and coagulation FVIII detection.

## 2. Experimental Section

### 2.1. Chemicals and Reagents

Calibration plasma for fibrinogen (STA^®^ Unicalibrator), quantitative determination reagent (thrombin and reference plasma) for fibrinogen by Clauss method (STA^®^ Fibrinogen 5), immuno-depleted plasma for factor VIII assay (STA^®^ Deficient VIII), determination reagent of activated partial thromboplastin time (APTT) (STA^®^ PTT Automate 5), calibration plasma for factor VIII assays (STA^®^ Unicalibrator), and detection buffer (STA^®^-Owren-Koller Buffer) were purchased from Stago Diagnosis Technology Ltd. (Beijing, China). All protein aliquots were stored at −80 °C, thawed at 25 °C . Milli-Q water was used to prepare all protein solutions. The diluted protein aliquots were stored at 4 °C till the prompt use. Other chemicals employed were of analytical reagent grade.

### 2.2. Patients and Plasma Samples

One hundred and twenty plasma samples without any abnormal color or turbidity were collected from 120 patients (57 male, 63 female; mean age 42.3 years) at Southwest Hospital (Chongqing, China) in October 2011. Patients who were receiving heparin treatment were excluded from this study. One part of 109 mM sodium citrate was thoroughly mixed with nine parts of fresh venous blood. All plasma samples were detected by both QCM biosensor and optical coagulometry method for comparative analysis.

### 2.3. Fabrication of QCM biosensor

The quartz crystals used in this study were AT-cut with a basic resonance frequency of 10 MHz (Chinese Electronic Scientific and Technical Group Company, Chongqing, China). The crystal consists of a 13-mm diameter and 0.15-mm thickness quartz wafer attached with two Ag electrodes (4 mm in diameter). The QCM biosensor, including electronic oscillation circuit, voltage stabilizer, thermal control system and 2 × 5 detection cells, was made by the Jialing Group (Chongqing, China).

The surfaces of the quartz crystals were spin-coated with polystyrene (0.5 wt% in chloroform) at 2,500 rpm for 5 min to make hydrophobic surfaces which is favorable to fibrinogen absorption. Afterwards, the crystals were thoroughly washed with Milli-Q water and dried with nitrogen to obtain a clean surface. The 2 × 5 model detection cells were assembled with the cleaned crystals.

### 2.4. Optical Coagulometry

The optical coagulometer, a STAGO-ST4 automatic analysis instrument (Diagnostic Stago, Asnsieres, France), was used to compare concentration of fibrinogen and FVIII in the plasma specimens with the QCM biosensor. The optical experiments were carried out in reaction cuvette with a steel bead vibrated by an alternating magnetic field. The citrated plasma specimens with various concentrations of FVIII were poured into the reaction cuvette, an equivalent volume of APTT reagent solution was mixed in succession. The time was counted as an aliquot part of 0.025 M CaCl_2_ solution was added into the cuvette. When the vibration of the steel bead stopped owing to the plasma coagulation, the concentration of FVIII could be obtained from the APTT of the tested plasma. The fibrinogen concentration of plasma specimens was detected with quantitative determination reagent of fibrinogen by the same method.

### 2.5. QCM Detection Test

The whole detection procedure was monitored in real time by the QCM biosensor and the frequency changes of the crystals were recorded and displayed on a computer. The polystyrene-coated quartz crystals were installed into the QCM detection chamber. The temperature of the system was maintained at 37 °C and relative humidity was controlled at approximately 60% based on our pilot experiments by a humidifier (FE-KXF 15, Panasonic, Japan). The oscillation frequency of the quartz crystals stabilized about 5 min after infusion of 100 μL reaction buffer and an essential resonance frequency was recorded. Thereafter quantitative determination reagent and calibration plasma were injected sequentially onto the surface of crystals. The resonance frequency during the plasma addition was recorded in real time. The gate time for the oscillation frequency measurement was 1,000 s, with a sampling period of 0.1 s.

#### 2.5.1. Measurement Procedure of Fibrinogen

**Figure 1.**
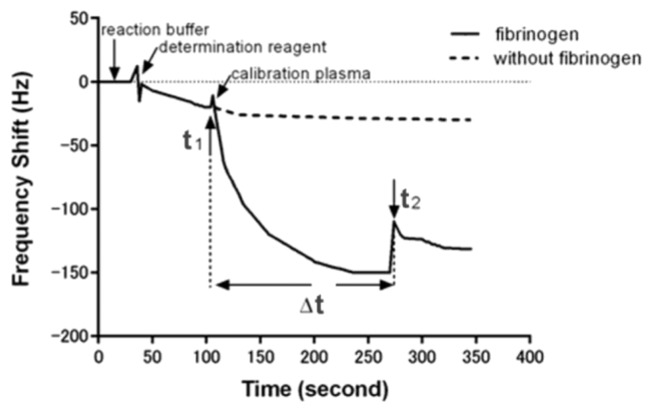
The real-time QCM biosensor detection diagram of the fibrinogen.

Briefly, 60 μL of quantitative determination reagent of fibrinogen was first added into the detection cell. When an initial baseline of frequency which corresponds to the addition of determination reagent was obtained, 20 μL calibration plasma of fibrinogen at different concentrations was added. An immediate sharp frequency decrease occurred with the exception of a short up rush caused by the influence of sample fluid, the time of calibration plasma adding was recorded as start-point (t_1_). The change in frequency leveled off after about 3 min, and a suddenly frequency change caused by plasma coagulation happened within 5 min and was recorded as end-point (t_2_). Thus, the clotting time which was caused by the plasma coagulation reaction can be calculated by the start-point and end-point (Δt = t_2_ − t_1_). A subsequent rinse with PBS did not induce significant frequency change, indicating an irreversible adsorption. The frequency shift of each step for monitoring the detecting fibrinogen was shown in Figure 1.

#### 2.5.2. Measurement Procedure of Factor VIII Detection

The measurement of factor VIII was performed as follows: first, 60 μL of mixture solutions containing 20 μL plasma immunodepleted for factor VIII, 20 μL determination reagent of APTT and 20 μL calibration plasma for factor VIII assays were poured into an Eppendorf tube. After blending for 60 s at room temperature, 20 μL of the produced mixture was incubated in the detection cell until a stable frequency was obtained. Then, the coagulation reaction was initiated by addition of 10 μL of 0.025 mol/L CaCl_2_ to the detection cell. The time-point of CaCl_2_ addition was taken as start-point in the data evaluation (t_1_). The propagation of the coagulation was observed as an initial lag time followed by a suddenly frequency shift (end-point, t_2_). The end-point describes the viscosity change of the plasma caused by coagulation reaction. After the coagulation reaction is completed, the clotting time can be analyzed to attain a description of the coagulation reaction. The measurements for fibrinogen and factor VIII were repeated three times to provide stable standard curves and confirmative output.

## 3. Results and Discussion

### 3.1. Correlation between Frequency Response and Viscosity Change

During blood coagulation, the coagulation reaction changes the viscosity of the plasma, and then changes the frequency of the QCM. The frequency changes of the QCM biosensor reflect not only the quality change, but also variations in the density and viscosity changes of the system. In the coagulation reaction, thrombin act on fibrinogens, fibrinogens decompose into peptide compounds and fibrin, then fibrins gather and form insoluble clots. Changes in the viscosity of plasma were caused by the formation of the fibrin clot. On the other hand, the viscosity change shifts the resonance frequency of the QCM biosensor enabling real-time continuous monitoring of this biological event. The viscosity of the reaction system changed instantaneously during the coagulation formation, and the mass deposition on the surface of crystal also changed. Both of them caused the sudden frequency change of the crystal, so we can judge the end-point (t_2_) of the reaction easily. By monitoring the signal output, a distinct clotting time can be obtained. During the coagulation process, the frequency signal of the crystal change steadily over time. When the fibrin clot finally formed, there will be a significant change of viscosity and result in a significant frequency change. The significant changes of frequency signal indicate the endpoint of coagulation reaction, since there are no subsequent changes in viscosity that would result in a change in frequency signal. The detachment of clots from the quartz surfaces could be another factor which induces abrupt frequency at t_2_. However, the clot detachment should induce a more drastic frequency shift which brings the frequency back to the calibration plasma, the current frequency shift at t_2_ reflects the coagulation reaction instead of clot detachment. Thus, the frequency changes of the QCM biosensor accurately reflect the process of plasma agglutination reaction. We can calculate the coagulation time from the start-point (sample adding point, t_1_) and the end-point (t_2_).

Making use of the special properties of QCM biosensor and the viscosity change of the coagulation reaction, we proposed a new strategy for fast detection of fibrinogen and coagulation factor VIII in plasma specimens. The designed system demonstrated that the proposed QCM biosensor can be successfully used for accurate and fast detection. End-point of reaction, frequency change of the crystal and total coagulation time formed the basis of analysis.

### 3.2. Optimizing Conditions of the Experiment

#### 3.2.1. Optimizing Temperature Conditions

**Figure 2.**
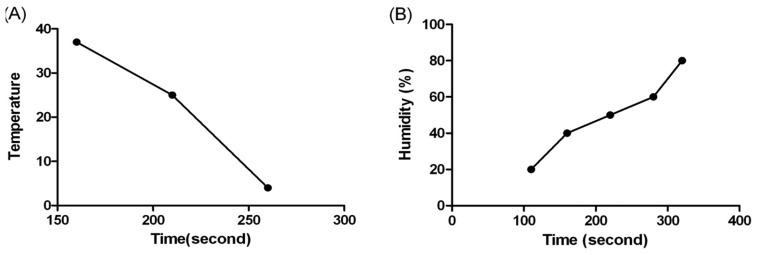
Optimization of QCM experiment conditions: (**A**) Reaction temperature dependent clotting time by QCM biosensor. (**B**) The effect of humidity on clotting time.

Temperature and humidity are two of the most important performance conditions which should be considered for the detection of coagulation reactions. The coagulation time varies with different temperature and humidity due to the effect of evaporation rate which changes the amount of water molecules in the samples. To further characterize the influence of temperature on the detection of coagulation time, the experiment was carried out at 4 °C, 25 °C and 37 °C, respectively. The humidity was set at 80% for all experiment to ensure full equilibrium of the reaction. As shown in Figure 2(A), the results of 4 °C showed a maximum duration, while the results of 37 °C gave a shortest coagulation time. We judged 37 °C to be the optimal temperature and used it in the rest of our study, since this temperature was also known as the physiological temperature of human body.

#### 3.2.2. Optimizing Humidity Conditions

Humidity also affects the evaporation rate severely, thus changing the coagulation reaction results. A series of humidity values was evaluated at 37 °C, and the results are shown in Figure 2(B). The coagulation time extended with the increase of humidity. The evaporation rate of the plasma decreased with higher humidity, which reflects a long coagulation time. The results revealed that the maximum coagulation time was obtained at about 80% humidity. Since 80% humidity is very rare in common environmental conditions, 60% humidity was chosen as the optimal condition for the experiments.

#### 3.2.3. Optimizing Calcium Chloride Concentration

**Figure 3.**
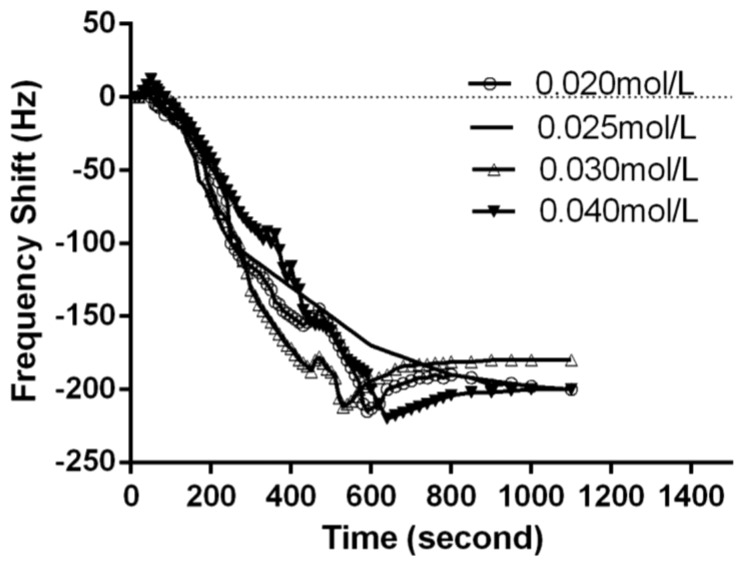
The effect of calcium chloride concentration on coagulation reaction.

Calcium is also required for the prothrombinase complexes to function. Calcium mediates the binding of the complexes via the terminal gamma-carboxy residues on factor Xa and factor IXa to the phospholipid surfaces expressed by platelets, as well as procoagulant microparticles or microvesicles shed from them [26]. For optimization of calcium chloride concentration, 0.020, 0.025, 0.030 and 0.040 mol/L of calcium chloride solution were used for the clotting time measurement. As shown in Figure 3, 0.025 mol/L calcium chloride gave a stable APTT value. Thus, 0.025 mol/L of calcium chloride solution was used for factor VIII detection throughout the following experiments.

### 3.3. Detection of Fibrinogen by QCM Biosensor

**Figure 4.**
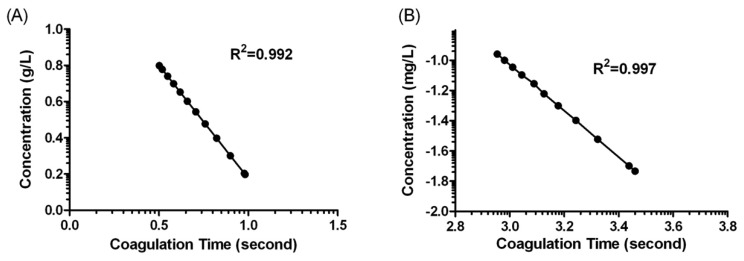
(**A**) The relationship between the log concentration of fibrinogen and log clotting time. (**B**) The relationship between the log concentration of coagulation factor VIII and log clotting time. Three replicate measurements per specimen were measured.

Figure 1 shows the behaviour of the oscillation frequency by the plasma sample with and without fibrinogen addition in the coagulation reaction. When 20 μL of the samples were dripped onto the crystals, the frequency decreased rapidly. This result could be attributed to the mass loading and the viscosity change on the interfacial layer of the quartz crystals. In the case of sample containing no fibrinogen, the frequency was kept in a constant value over the reaction duration. Whereas, the frequency would gradually drop to a relatively low value after the fibrinogen calibration plasma added. The descending phenomena of frequency lasted for about 180 s and then returned to a significant change because of the fibrin clot formed. We could conclude such a frequency response was induced by viscosity change which was caused by fibrinogen formation. In general, it would take 300 s for normal blood coagulation, owing to a series of cascade enzyme reactions. In fact, the coagulation time that the system reflected in frequency response was extremely dependent on the fibrinogen concentration present in the sample. The more fibrinogen the sample contained, the less coagulation time resulted owing to the fast coagulation reaction. The standard curve for fibrinogen was made by detecting different concentration of fibrinogen calibration plasma, and the correlation between coagulation time and fibrinogen concentration were statistically calculated as shown in Figure 4. There was a good linear correlation in a double-logarithmic plot of APTT *versus* fibrinogen concentration in the range of 1.58–6.30 g/L. The correlation equation was log C (g/L) = 1.427 – 1.251 × log t (s) with a correlation coefficient of 0.992.

### 3.4. Detection of Factor VIII by QCM Biosensor

The placement of a plasma sample onto the crystal surface of the QCM biosensor, and addition of Ca^2+^ to initiate the coagulation process, typically resulted in an obvious frequency shift. It can be seen that the clot time was shortened with the increased concentration of factor VIII. The decrease of clot time shows a dependency on the concentration of factor VIII, with the decrease of frequency occurring sooner and being steeper for the higher concentrations of factor VIII than for the lower concentrations. A linear relationship was observed in the range of 0.0185 – 0.111 mg/L concentration of factor VIII. The correlation equation was log C (mg/L) = 3.561 – 1.530 × log t (s) with a correlation coefficient of 0.997. All the experimental data were reproducible, showing a standard deviation that was less than 8%, which indicates good reproducibility.

### 3.5. Detection of Fibrinogen and FVIII in Plasma Specimens

We further tried fibrinogen and FVIII detection in clinical samples. The concentrations of fibrinogen and FVIII in clinical plasma specimens were simultaneously measured by the QCM biosensor and the STAGO-ST4 optical coagulometer. In 120 clinical plasma specimen cases, there was an excellent correlation between these two methods (R^2^ = 0.949 for fibrinogen and 0.948 for FVIII) over the shared dynamic range of QCM biosensor. The results are shown in Table 1. The mean concentrations of fibrinogen detected by QCM biosensor and optical coagulometer were (4.03 ± 0.32) g/L and (4.24 ± 0.23) g/L, respectively. The results suggest good consistency and clinical comparability between these two methods. This suggests that QCM biosensor could exhibit accurate and fast detection for fibrinogen and FVIII in clinical samples.

## 4. Conclusions

In the present work, we have developed a new method to detect fibrinogen and coagulation factor VIII in clinical plasma specimens with a QCM biosensor. Under the conditions of 37 °C temperature and 60 % humidity, fibrinogen and coagulation factor VIII can be detected quickly (within 10 min) by our QCM biosensor. The viscosity change and mass deposition characters of the coagulation reaction made an apparent step-ladder curve with the QCM biosensor that was distinctly suitable for determining the coagulation time. Real time monitoring and more convenient operation are its advantages compared with traditional manual methods. The results of fibrinogen and coagulation factor VIII detected by the QCM biosensor coincide well with those detected by the optical coagulometer. The QCM biosensor proved to be an accurate and fast method for fibrinogen and FVIII detection, while additional advantages of the QCM method are its low cost and simple instrumentation. On the basis of these results, the QCM biosensor can be a sensitive and effective method for studying viscosity properties of biopolymers and biological activities of plasma specimens. However, we have to point out that more detailed work should be done before its clinical application. Optical machines are vulnerable to interferences like hemolysis, icterus and lipemia that can cause unusual patterns in clotting curves. The effect of these interferences on QCM biosensor-derived fibrinogen detection should be examined in further studies. In the future, we hope to provide a portable, label-free device for the accurate and fast detection of plasma coagulation reaction.

## Figures and Tables

**Table 1.** Correlation between QCM biosensor and OC method for fibrinogen and factor VIII detection.

	**Fibrinogen (g/L)**		**Coagulation Factor VIII (mg/L)**	
	
	**QCM biosensor**	**OC**	**QCM biosensor**	**OC**
Mean	4.03	4.24	0.094	0.089
S.D.	0.32	0.23	0.09	0.08
CV	7.94%	5.42%	6.80%	5.21%
Correlation	0.949		0.948
